# Photobiomodulation Therapy Combined with a Static Magnetic Field Applied in Different Moments Enhances Performance and Accelerates Muscle Recovery in CrossFit® Athletes: A Randomized, Triple-Blind, Placebo-Controlled Crossover Trial

**DOI:** 10.1155/2022/9968428

**Published:** 2022-07-19

**Authors:** Henrique Dantas Pinto, Heliodora Leão Casalechi, Thiago de Marchi, Caroline dos Santos Monteiro Machado, Luana Barbosa Dias, Matheus Marinho Aguiar Lino, Jonatas Bezerra de Azevedo, Shaiane Silva Tomazoni, Ernesto Cesar Pinto Leal-Junior

**Affiliations:** ^1^Laboratory of Phototherapy and Innovative Technologies in Health (LaPIT), Postgraduate Program in Rehabilitation Sciences, Nove de Julho University, São Paulo, Brazil; ^2^University Center of Bento Gonçalves (UNICNEC), Bento Gonçalves, Rio Grande do Sul, Brazil; ^3^Physiotherapy Research Group, Department of Global Public Health and Primary Care, University of Bergen, Bergen, Norway; ^4^ELJ Consultancy, São Paulo, Brazil

## Abstract

The ergogenic effects of photobiomodulation therapy combined with a static magnetic field (PBMT-sMF) on exercises with characteristics similar to those of CrossFit® are unknown. This study was aimed at investigating the effects of PBMT-sMF applied at different times on recovery and physical performance in CrossFit® athletes by analyzing functional aspects, muscle damage, inflammatory processes, and oxidative stress. This was a prospectively registered, triple-blinded, placebo-controlled, crossover trial. CrossFit® athletes were recruited and assigned to receive one of the four possible interventions. Each intervention included protocols before and after the exercise (referred to as the workout of the day (WOD)). The four possibilities of intervention were as follows: placebo before and after WOD (placebo), PBMT-sMF before and placebo after WOD (PBMT-sMF before), placebo before and PBMT-sMF after WOD (PBMT-sMF after), and PBMT-sMF before and after WOD (PBMT-sMF before and after). The order of possibilities for the interventions was randomized. The primary outcome was the functional test performance. The secondary outcomes were the subjective perception of exertion, muscle damage, inflammation, and oxidative stress. The outcomes were measured before the WOD; immediately after the intervention; and 1, 24, and 48 hours after the WOD. Statistical analysis was performed using repeated measures ANOVA followed by the Bonferroni post hoc test to examine the differences between the interventions at each time point. Twelve participants were randomized and analyzed for each sequence. PBMT-sMF enhanced the performance on functional tests (calculated as a percentage of change) when applied before or after WOD in the assessment performed immediately post-WOD and at 24 and 48 hours later (*p* < 0.05) compared to placebo and PBMT-sMF before and after WOD. In terms of the secondary outcomes, PBMT-sMF applied before or after WOD significantly decreased the creatine kinase, catalase, and superoxide dismutase activities and interleukin-6, thiobarbituric acid, and carbonylated protein levels (all *p* < 0.05) compared to the other possibilities of intervention. In addition, PBMT-sMF applied before and after WOD decreased creatine kinase activity at 24 hours and IL-6 levels at 24 and 48 hours compared to placebo (*p* < 0.05). None of the participants reported any adverse events. PBMT-sMF enhanced the performance of functional tests, decreased the levels of biochemical markers of muscle damage and inflammation, decreased oxidative stress, and increased antioxidant activity in CrossFit® athletes when applied before or after WOD.

## 1. Introduction

CrossFit® was introduced in the early 2000s as a strength-training and conditioning system [1, 2]. The training routine combines constantly varying functional and high-intensity movements [3]. These workouts are called the workout of the day (WOD) and consist of a wide range of movements, including powerlifting, Olympic weight lifting, gymnastic movements, calisthenics, and cardiorespiratory endurance [4]. WODs are scored and often involve competition between participants [2] and are usually performed with fast and successive repetitions with limited or no recovery time between sessions [5]. Therefore, these competitions involve physically demanding activities, with high demands on the anaerobic energy system. As a result, substantial metabolic and muscular fatigue and oxidative stress develop, decreasing physical performance [6]. Considering that mitigating the damage caused by intense exercise is needed, increasing performance, accelerating recovery between sessions, and using therapeutic agents with ergogenic effects can be important allies to CrossFit®.

Photobiomodulation therapy (PBMT) is a therapeutic agent with ergogenic effects that can be used in CrossFit® athletes. PBMT is a light therapy that uses nonionizing light sources such as lasers, LEDs, and broadband light (usually low power, with an output of 1–500 mW and density of 1 mW–5 W/cm^2^) from the visible to the infrared spectrum (600–1000 nm wavelength) [7]. Static magnetic field (sMF) associated with PBMT (PBMT-sMF) reportedly generates greater electron transfers, thereby activating the mitochondrial respiratory chain and increasing the production of adenosine triphosphate [8]. Therefore, PBMT and sMF have been used in combination with the same device to achieve greater positive effects [9]. PBMT as a single intervention or PBMT-sMF promotes ergogenic effects in different sports and physical training [9–11], accelerates muscle recovery, and enhances postexercise performance [10]. In these cases, PBMT alone or in combination with sMF decreases muscle damage [10] and oxidative stress and modulates inflammation [11]. In addition, PBMT-sMF positively affects the physiological response to physical exercise, contributing to muscle function recovery [10].

PBMT-sMF accelerates postexercise recovery and enhances physical performance in different types of exercise and at different treatment moments [10]. PBMT-sMF promotes ergogenic effects when applied before and after endurance training and before strength training [10]. However, to date, it is unknown whether PBMT-sMF can decrease the recovery time between sessions and enhance athletes' performance in exercises with characteristics similar to those of CrossFit®, which include exercises that involve strength, aerobic and muscular resistance, high-intensity, fast, and successive repetitions, and no or limited recovery/pause time. In addition, the best moment to apply PBMT-sMF for maximum recovery and enhancement of physical performance in athletes performing activities similar to CrossFit® athletes remains undetermined. Furthermore, evidence supporting PBMT-sMF as a redox intervention to accelerate postexercise recovery and enhance physical performance is limited, and its use in the CrossFit® field is still unknown.

Therefore, this study was aimed at investigating the effects of PBMT-sMF applied at different time points (before, after, or before and after WOD) on recovery and physical performance in CrossFit® athletes by analyzing functional aspects, muscle damage, inflammation, and oxidative stress.

## 2. Materials and Methods

### 2.1. Study Design and Ethics Statements

A randomized, triple-blind (outcome assessors, participants, and therapists), placebo-controlled crossover trial was performed. The study was carried out in four separate stages, one stage per week, with a washout period of seven days. The allocation ratio was 1 : 1 : 1 : 1. This trial was approved by the Research Ethics Committee of Nove de Julho University (process 3.360.743), and the protocol was prospectively registered on ClinicalTrials.gov (NCT04349085). All eligible participants were informed of the study procedures, and written informed consent was obtained before commencing with the study. No deviations from the registered protocols were observed.

### 2.2. Participants

Twelve participants were recruited from a CrossFit® box located in São José dos Campos, São Paulo, Brazil. The inclusion criteria were as follows:

- Healthy amateur CrossFit® athletes
- Athletes aged between 18 and 35 years
- Male athletes
- Athletes who had been practicing CrossFit® for at least 1 year
- Athletes who voluntarily participated in all stages of the trial

The exclusion criteria were as follows:

- History of musculoskeletal injury such as muscle strain, contusion, and sprain in the hips, knees, and calves in the month preceding the trial
- Use of any pharmacological agents
- Occurrence of musculoskeletal or joint injuries, such as muscle strain, contusion, sprain, and fracture during the trial

### 2.3. Randomization

The 12 participants and interventions were randomized. Thus, all participants underwent the four interventions in different weeks with a washout period of 7 days. The possibilities of the interventions were as follows: (1) placebo, (2) PBMT-sMF before WOD, (3) PBMT-sMF after WOD, or (4) PBMT-sMF before and after WOD. We generated codes through a website (random.org) to ensure that, in the first stage, each intervention involved 25% of the participants. For the succeeding stages, 25% of the participants were involved in each of the four interventions to ensure equal distribution of participants among the four interventions during the four stages of the trial. Randomization was performed by a researcher who was not involved in the assessment of the participants or the application of the interventions. Another researcher who did not participate in any stage of data collection or analysis was responsible for programming the PBMT-sMF device into active or placebo mode and was instructed not to disclose the program until the study was completed. Concealed allocation was achieved by using sequentially numbered and sealed opaque envelopes.

### 2.4. Blinding

The PBMT-sMF device used was previously programmed in active or placebo mode. Therefore, the outcome assessors, participants, and therapists who applied the intervention and guided the CrossFit® sessions were blinded throughout the treatment. During irradiation, the device displayed the same sounds, lights, and information, regardless of the programmed mode, ensuring the blinding of the therapist and participants. To maintain blinding, participants wore opaque goggles to prevent them from seeing whether light was being irradiated.

### 2.5. Interventions

PBMT-sMF (active or placebo) was applied in two steps at all stages of the workout: 5 min before the WOD and 5 min after the WOD. Based on randomization, the participants received one of the four interventions daily, exhausting the different possibilities in the fourth stage. The four intervention possibilities are as follows:

1. Placebo: placebo before WOD and placebo after WOD (placebo+placebo)
2. PBMT-sMF before WOD: active PBMT-sMF before WOD and placebo after WOD (PBMT-sMF+placebo)
3. PBMT-sMF after WOD: placebo before WOD and active PBMT-sMF after WOD (placebo+PBMT-sMF)
4. PBMT-sMF before and after WOD: active PBMT-sMF before and after WOD (PBMT-sMF+PBMT-sMF)

PBMT-sMF (active or placebo) was applied with the device in direct contact with the participant's skin and light pressure at four sites in the knee extensor/hip flexor muscles, three sites in the knee flexor/hip extensor muscles, and one site in the plantar flexor muscles in both the lower limbs. For the application of PBMT-sMF, a cluster probe with 20 diodes was used (four diodes of 905 nm (1.25 average power of peak power 50 W for each diode), eight diodes of 850 nm (average power of 40 mW for each diode), and eight diodes of 633 nm (average power of 25 mW for each diode)), which was manufactured by Multi Radiance Medical® (Solon, OH, USA). The active PBMT-sMF dose was approximately 270 J, 180 J, and 60 J for knee extensors/hip flexors, knee flexors/hip extensors, and plantar flexors, respectively. Doses were selected based on the therapeutic window, as evidenced by a recent systematic review [10]. Moreover, the doses used for each muscle group were previously optimized for the technology based on several studies carried out by our research group [9]. The active PBMT-sMF parameters are listed in Table 1. The placebo treatment was performed using the same device as the active PBMT-sMF and, in the same way, using the same application times and sites. However, in the placebo mode, the infrared laser diodes, infrared LED diodes, and sMF were deactivated (turned off), and the power of the red LED diodes was decreased to 0.5 mW (mean power for each diode) to ensure that a red light was still visible but without delivering an effective therapeutic or considerable dose (maximum of 1.38 J per muscle group).

### 2.6. Procedures

The study was conducted in four separate stages: one stage per week, with an interval of seven days between them, for a total of four weeks. At each stage, the volunteers performed the WOD (exercise protocol) and received the intervention protocol according to previous randomization. Volunteers were assessed before and after WOD at each stage.

#### 2.6.1. Exercise Protocol: WOD

To control for potential variation related to the circadian cycle, all participants performed the exercise protocol (WOD) at the same hour of the day for each of the four interventions. Participants were requested to refrain from any physical activity until the end of the last assessment at 48 hours after the WOD. The aim was to finish the WOD thrice in a well-known series model for athletes (21-15-9 model) without rest and as quickly as possible. As a result, in the first series, the athletes completed twenty-one (21) repetitions; in the second, the number of repetitions dropped to fifteen (15); and in the last, it dropped to nine (9) repetitions. During WOD, an assessor counted the number of repetitions and checked their validity. The sequence of the three exercises was as follows.

Calories in the Assault AirBike®: the athletes pedaled the bicycle until they reached the required number of calorie repetitions. The most popular model, similar to that used in CrossFit® Games, was used.

Hang squat clean: this exercise was started with the athlete standing with the body completely extended and holding a barbell weighing 40 kg close to the body. The athlete then threw the barbell up to the shoulders while performing a full squat, breaking the parallel line of the patella relative to the iliac crest in the deep phase of the movement. The athlete ended the movement by extending his body again.

Box jump over: in this exercise, the athlete jumped on top of a box with a height of 60.69 cm (24 in), touched the top of the box with both feet, and jumped to the ground on the opposite side.

### 2.7. Outcomes

The primary outcome was a change in performance on a functional test. The secondary outcomes were the subjective perception of exertion, muscle damage, inflammation, and oxidative stress. Data on these outcomes were collected by an assessor blinded to the allocation of the participants to their intervention schedule. The procedures for outcome assessments were as follows.

Performance on the functional test was measured by the maximum number of free squat repetitions in 1 minute.

We used a simple free squat test as an indicator of performance on the functional test. We instructed the participants to perform the maximum number of repetitions within one minute. The assessor counted the number of valid measurements. An invalid repetition was defined as any in which the participant either did not break the parallel line of the patella in relation to the iliac crest in the deep phase of the movement or did not finish the movement with complete knee and hip extension on initial movement. The maximum number of free squat repetitions was measured before (baseline), immediately after intervention, and 1, 24, and 48 hours after the WOD. The percentage change was calculated based on baseline values.

Exertion subjective perception was measured in terms of rating of perceived exertion (RPE).

The CR-100 Effort Perception Scale was used as an internal load indicator. This scale is simple, noninvasive, free of charge, and validated for measuring the perception of exercise intensity [12–14]. It is also considered more accurate than the CR-10 scale [15, 16]. To differentiate between the perceived effort in the lower limb musculature and cardiorespiratory capacity, the participants were asked to assign scores for leg fatigue (RPE-MI) and cardiorespiratory fatigue (RPE-R) from 0 to 100. For scoring, two identical scales were presented to the participant so that he could quantify his condition at that time. All details regarding the completion and interpretation of the scale were provided before any procedure. The researchers ensured that each participant understood the study procedure. Subjective perception of exertion was measured before (baseline), immediately after intervention, and 1, 24, and 48 hours after the WOD.

Muscle damage, inflammation, oxidative stress, and antioxidant activity were measured by assessing blood samples.

Blood samples measuring 5 ml were collected from the anterior cubital vein to analyze muscle damage, inflammation, and oxidative stress before (baseline) and 1, 24, and 48 hours after the WOD. These time points were chosen because muscle damage and the inflammatory process peak over a time course of up to 48 hours after physical exercise [9]. One hour after each sample was obtained, the samples were centrifuged at 3000 rpm for 20 min. The serum was stored in tubes (Eppendorf®) at -80°C for further analysis. The activity of creatine kinase (CK) was analyzed as an indirect marker of muscle damage by spectrophotometry using specific reagent kits manufactured by Labtest® (Minas Gerais, Brazil). The interleukin- (IL-) 6 level was analyzed as an inflammatory marker using enzyme-linked immunosorbent assay kits manufactured by BD® (New Jersey, USA), according to the manufacturer's instructions. Thiobarbituric acid reactive substance (TBARS), carbonylated protein, catalase (CAT), and superoxide dismutase (SOD) activities were analyzed to measure oxidative stress and antioxidant activity. TBARS, carbonylated protein, CAT, and SOD activities were analyzed using spectrophotometry and specific reactions previously described [17–20]. All analyses were performed in triplicate. The median value was used for statistical analysis.

### 2.8. Characterization of the Sample

To date, no studies have analyzed the effects of PBMT-sMF on performance and postexercise recovery in CrossFit® athletes. Hence, the number of participants was calculated based on the results obtained in the first week (phase) of this study. The calculation was performed with 12 participants (three athletes per treatment) by a researcher who was not involved in data collection and who was unaware of the allocation of the participants. In this first week/phase, when PBMT-sMF was applied before the WOD, the percentage change (from baseline) in the number of squats (primary outcome of this study) performed immediately after the treatments was 98.45% (standard deviation, 7.28); when applied after the WOD, the percentage of change immediately after the treatments was 99.01% (standard deviation of 8.11); when applied before and after the WOD, the percentage of change immediately after the treatments was 97.92% (standard deviation of 6.53); and when placebo PBMT-sMF was applied before and after the WOD, the percentage of change was 90.88% (standard deviation of 7.57).

The means and standard deviations resulted in an overall effect size of 1.2171 (calculated using the online tool available at https://webpower.psychstat.org/models/means03/effectsize.php). Considering an *α* of 5% and using the ANOVA test, a sample of 12 volunteers per treatment/moment tested resulted in a statistical power of 80% (*β* value of 20%). The calculations were performed using the Statistics Kingdom website (https://www.statskingdom.com/sample_size_regression.html).

Since this trial was a crossover study, this was the total sample size.

### 2.9. Statistical Analysis

Statistical analysis was conducted following intention-to-treat principles [21, 22]. The researcher who performed the statistical analysis was blinded to the randomization and allocation of the volunteers to the experimental groups. We first tested the data for normal distribution using the Shapiro-Wilk test. Since the data showed a normal distribution for both primary and secondary outcomes, we performed a repeated measures ANOVA followed by the Bonferroni post hoc test to test between interventions and the differences at each time point. We analyzed the data in terms of both their absolute values and percentage of change based on the values established from the baseline tests. The significance level was set at *p* < 0.05. Data are presented as mean ± standard deviation (SD) in the tables. For the graphical data, the mean and standard error of the mean (SEM) were used.

## 3. Results

**Figure 1:**
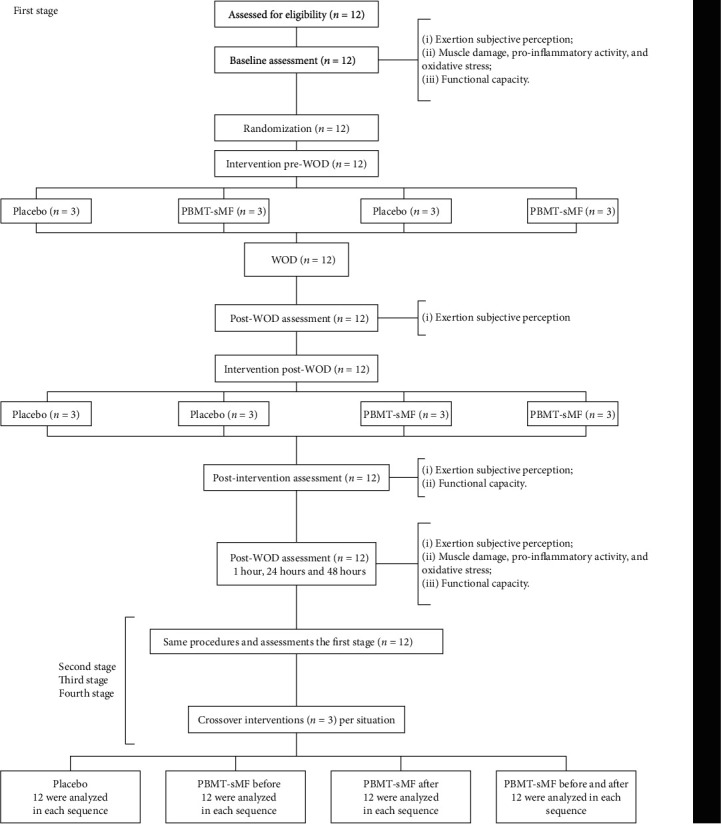
Enrollment and randomization.

Twelve CrossFit® athletes with an average age of 27 years (±5.54), average height of 176 cm (±5.6), and average weight of 81.5 kg (±3.9) were randomized. All of them completed all procedures. The results were analyzed for each sequence between April and May 2020 (Figure 1).

There was no evidence of learning effects regarding this protocol, since over the four weeks of the study, there was no statistically significant difference (*p* > 0.05) in the outcomes regarding the length of the WODs, regardless of the intervention. In the first week, the average time was 514.90 sec (±77.52); in the second week, 486.50 sec (±67.43); in the third week, 489.80 sec (±94.39); and in the fourth week, 474.50 sec (±106.60).

**Figure 2:**
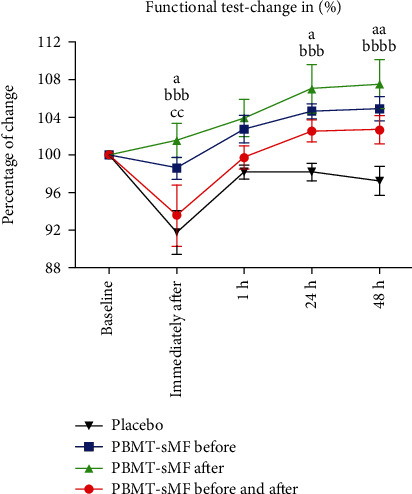
Percentage of change in number of squats performed. The data are presented in mean and SEM. ^a^PBMT-sMF before WOD compared to placebo (*p* < 0.05); ^aa^PBMT-sMF before WOD compared to placebo (*p* < 0.01); ^bbb^PBMT-sMF after WOD compared to placebo (*p* < 0.001); ^bbbb^PBMT-sMF after WOD compared to placebo (*p* < 0.0001); and ^cc^PBMT-sMF after WOD compared to PBMT-sMF before and after WOD (*p* < 0.01).

Figure 2 shows the percentage change in performance in the functional test. There was a significant difference in the PBMT-sMF after WOD compared to that in placebo (*p* = 0.0001) and PBMT-sMF before and after WOD (*p* = 0.0030) in the assessment immediately after the second step of intervention (Figure 2). There was also a significant difference between PBMT-sMF after WOD and placebo in the assessment at 24 h (*p* = 0.0007) and 48 h (*p* < 0.0001) after WOD (Figure 2). Finally, there was a significant difference between PBMT-sMF before the WOD and placebo in the assessments performed immediately after the second step of intervention (*p* = 0.0168) and 24 h (*p* = 0.0279) and 48 h (*p* = 0.0047) after the WOD (Figure 2).

**Figure 3:**
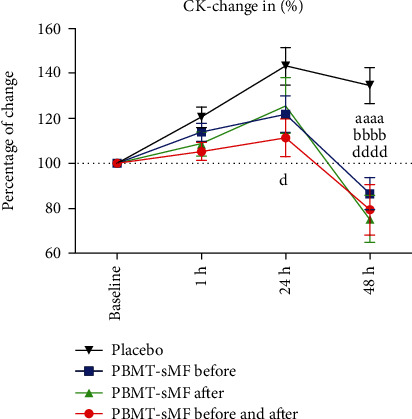
Percentage of change in CK activity. The data are presented as mean and SEM. ^aaaa^PBMT-sMF before WOD compared to placebo (*p* < 0.0001); ^bbbb^PBMT-sMF after WOD compared to placebo (*p* < 0.0001); and PBMT-sMF before and after WOD compared to placebo (*p* < 0.0001).

Figure 3 shows the percentage change in CK activity. There was no difference between the moments at which PBMT-sMF was applied (before, after, or before and after) at all the time points tested (*p* > 0.05). However, there was a significant difference between PBMT-sMF before and after WOD and placebo treatment at 24 h (*p* = 0.0101) (Figure 3). There was also a significant difference in the PBMT-sMF before WOD, PBMT-sMF after WOD, and PBMT-sMF before and after WOD compared to placebo (*p* < 0.0001) at 48 h after WOD (Figure 3).

**Figure 4:**
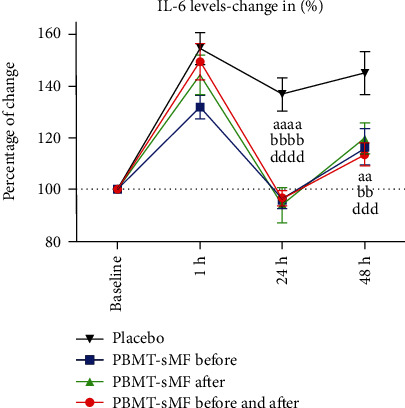
Percentage of change in IL-6 levels. The data are presented as mean and SEM. ^aa^PBMT-sMF before WOD compared to placebo (*p* < 0.01); ^aaaa^PBMT-sMF before WOD compared to placebo (*p* < 0.0001); ^bb^PBMT-sMF after WOD compared to placebo (*p* < 0.01); ^bbbb^PBMT-sMF after WOD compared to placebo (*p* < 0.0001); ^ddd^PBMT-sMF before and after WOD compared to placebo (*p* < 0.001); ^dddd^PBMT-sMF before and after WOD compared to placebo (*p* < 0.0001).

Figure 4 shows the percentage change in the IL-6 levels. There was no difference between the moments at which PBMT-sMF was applied (before, after, or before and after) at all time points tested (*p* > 0.05). However, there was a significant difference between PBMT-sMF before WOD and placebo in the assessment of 1 h after WOD (*p* = 0.0187) (Figure 4). There was also a significant difference in the PBMT-sMF before WOD, PBMT-sMF after WOD, and PBMT-sMF before and after WOD compared to placebo (*p* < 0.0001) at 24 h (Figure 4). Furthermore, there was a significant difference in the PBMT-sMF before WOD (*p* = 0.0012), PBMT-sMF after WOD (*p* = 0.0059), and PBMT-sMF before and after WOD (*p* = 0.0003) compared to placebo at 48 h after WOD (Figure 4).

**Figure 5:**
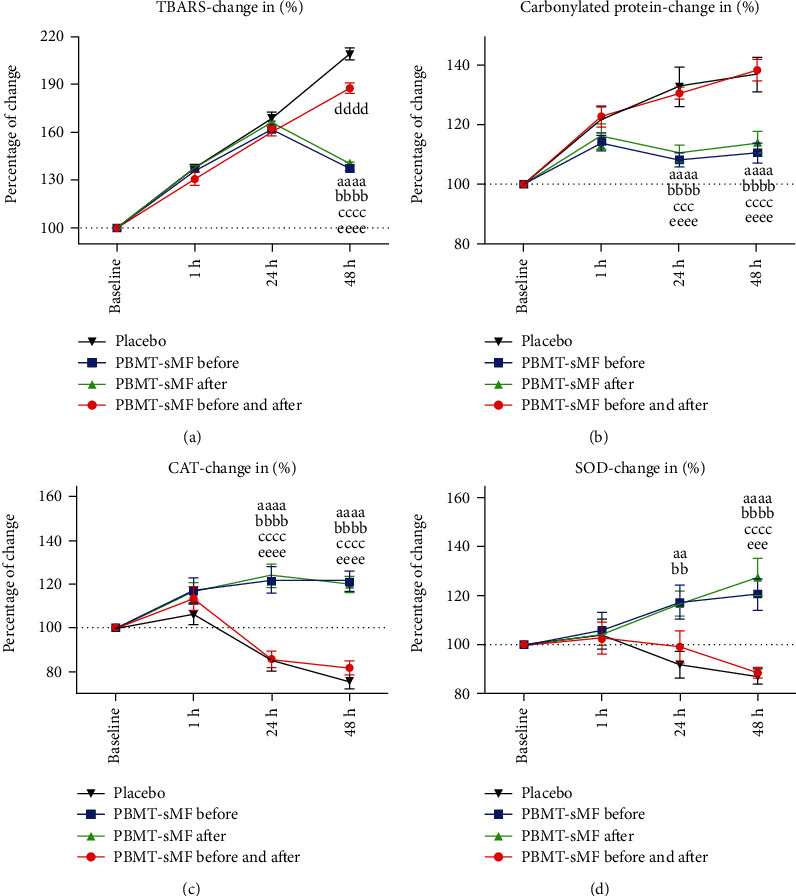
Percentage of change in TBARS, carbonylated proteins, CAT, and SOD. The data are presented as mean and SEM. ^aa^PBMT-SMF before WOD compared to placebo (*p* < 0.01); ^aaaa^BMT-sMF before WOD compared to placebo (*p* < 0.0001); ^bb^PBMT-sMF after WOD compared to placebo (*p* < 0.01); ^bbbb^PBMT-sMF after WOD compared to placebo (*p* < 0.0001); ^ccc^PBMT-sMF after WOD compared to PBMT-sMF before and after WOD (*p* < 0.001); ^cccc^PBMT-sMF after WOD compared to PBMT-sMF before and after WOD (*p* < 0.0001); ^dddd^PBMT-sMF before and after WOD compared to placebo (*p* < 0.0001); and ^eeee^PBMT-sMF before WOD compared to PBMT-sMF before and after WOD (*p* < 0.0001).

Figure 5 shows the percentage changes in TBARS (Figure 5(a)), carbonylated proteins (Figure 5(b)), CAT (Figure 5(c)), and SOD (Figure 5(d)). For TBARS (Figure 5(a)), there was no difference in the percentage change between interventions in the assessments at 1 h and 24 h. In contrast, there was a significant difference in the PBMT-sMF before WOD and PBMT-sMF after WOD compared to placebo (*p* < 0.0001) and PBMT-sMF before and after WOD (*p* < 0.0001) at 48 hours. Moreover, there was a significant difference between PBMT-sMF before and after WOD and placebo treatment (*p* < 0.0001) at 48 hours.

Regarding the carbonylated proteins (Figure 5(b)), there was no difference in the percentage change between the interventions at 1 h. In contrast, there was a difference in the PBMT-sMF before WOD compared to placebo (*p* < 0.0001) and PBMT-sMF before and after WOD (*p* < 0.0001) in the 24 h assessment. There was also a significant difference in the PBMT-sMF after WOD compared to placebo (*p* < 0.0001) and PBMT-sMF before and after WOD (*p* = 0.0003) at 24 hours. Furthermore, there was a significant difference in the PBMT-sMF before WOD and PBMT-sMF after WOD compared to placebo (*p* < 0.0001) and PBMT-sMF before and after WOD (*p* < 0.0001) at 48 h (Figure 5(b)).

Regarding CAT (Figure 5(c)), there was no difference in the percentage change between the interventions in the assessment of 1 hour. In contrast, there was a significant difference in the PBMT-sMF before WOD and PBMT-sMF after WOD compared to placebo (*p* < 0.0001) and PBMT-sMF before and after WOD (*p* < 0.0001) at 24 and 48 h (Figure 5(c)).

For SOD (Figure 5(d)), there was no difference in the percentage change between the possibilities of intervention in the assessment of 1 h. In contrast, there was a significant difference in the PBMT-sMF before WOD and PBMT-sMF after WOD compared to placebo (*p* < 0.01) at 24 h. There was also a significant difference in the PBMT-sMF before WOD compared to placebo (*p* < 0.0001) and PBMT-sMF before and after WOD (*p* < 0.001) at 48 h. Furthermore, there was a significant difference in PBMT-sMF after WOD compared to placebo (*p* < 0.0001) and PBMT-sMF before and after WOD (*p* < 0.0001) at 48 h (Figure 5(d)).

None of the participants reported adverse events. In addition, there was no difference between the interventions at baseline for any of the outcomes analyzed. This again demonstrated that there were no learning effects or cumulative effects, given that this was a crossover study (Table 2). All absolute values for performance on the functional test, subjective perception of exertion, muscle damage, inflammation, and oxidative stress are summarized in Table 2.

## 4. Discussion

This is the first randomized, triple-blind, placebo-controlled trial to assess the effects of PBMT-sMF applied at different time points (before, after, or before and after WOD) on recovery and physical performance in CrossFit® athletes by analyzing functional aspects, muscle damage, inflammatory activity, and oxidative stress. We observed that PBMT-sMF enhanced the performance on functional tests when applied both before and after WOD in the assessment performed immediately after the second step of intervention and at 24 and 48 h post-WOD. PBMT-sMF decreased CK activity at 24 h when applied before and after WOD and at 48 h when applied at any moment (before or after WOD, in addition to before and after WOD). Furthermore, PBMT-sMF decreased the levels of IL-6 at 1 h (when applied before the WOD) and at 24 and 48 h when applied at any moment (before or after WOD, in addition to before and after WOD). Finally, PBMT-sMF decreased oxidative stress when used before or after WOD, mainly at 24 and 48 h. In contrast, PBMT-sMF applied before and after WOD did not decrease oxidative stress.

The WOD used in our study was developed to ensure that athletes performed movements under the highest possible intensity to generate fatigue, decrease maximum strength or power output [23], and lead to oxidative stress [24]. As previous studies have suggested, WODs by time proved to be more physically demanding than WODs, whose objective was to perform a greater number of repetitions in a fixed time [6]. Likewise, WODs with weight lifting exercises have a greater physiological response than those without weight lifting exercises [25]. Our results suggest that the WOD used in our study was successful, as the subjective and objective outcomes measured showed respiratory and muscle fatigue after WOD. Therefore, the observed results regarding the effects of PBMT-sMF on the recovery and physical performance of CrossFit® athletes can be considered reliable.

In recent studies, PBMT alone and PBMT-sMF have been shown to decrease the levels of biomarkers, such as CK and IL-6, which are related to muscle damage [10] and inflammatory processes [11], as well as attenuate the oxidative stress induced by exercise [11]. Furthermore, there is robust evidence that PBMT alone and PBMT-sMF act as ergogenic agents, accelerating postexercise recovery and enhancing physical performance [10]. These positive effects were observed in different modalities of exercise and protocols, such as repeated, isometric, and eccentric contractions; aerobic and strength training; and even field tests [10]. Our results corroborate this previous evidence [10, 11] since we observed that PBMT-sMF enhanced the performance of functional tests and modulated muscle damage and inflammation after WOD. Therefore, our results suggest that PBMT-sMF has effects similar to those previously observed in exercise modalities that combine strength, aerobic endurance, and muscular resistance, such as CrossFit®.

Evidence supporting the ergogenic effects of PBMT-sMF is robust [10]. However, the mechanism of action underlying these effects is still not well established. In recent years, there has been much discussion that PBMT or PBMT-sMF may enhance performance and accelerate postexercise recovery through the upregulation of antioxidant activity, which could lead to a decrease in exercise-induced oxidative stress. However, a previous study did not observe any modulation of CAT activity with PBMT use in nonathletic volunteers [26]. In addition, another study did not observe modulation of CAT and SOD with PBMT in high-level soccer players [27]. In contrast, in our study, we observed that PBMT-sMF decreased carbonylated protein production and lipid peroxidation related to oxidative damage to lipids and proteins and upregulated antioxidant activity. These findings are similar to the results of a previous study in which PBMT, with optimized parameters, decreased the levels of TBARS and carbonylated proteins and increased CAT and SOD activities in high-level soccer players [11]. Therefore, although evidence on the effects of PBMT or PBMT-sMF on oxidative stress is still divergent and scarce, our results corroborate the findings of Tomazoni et al. [11], suggesting that PBMT and PBMT-sMF have a potential antioxidant effect in athletes, which may be the key mechanism of action.

The moment PBMT or PBMT-sMF is applied (before and/or after exercise) is crucial for better results [10]. It is recommended that for a single application, PBMT or PBMT-sMF must be applied between 5 minutes and 6 hours before exercise. In addition, PBMT or PBMT-sMF should be applied immediately before each exercise session to gain strength. Finally, the recommendation for endurance training is that PBMT or PBMT-sMF irradiation must be performed immediately before and after each exercise session [10]. To date, the best time to apply PBMT-sMF in exercise modalities with characteristics similar to those of CrossFit® is unknown. In our study, we observed that PBMT-sMF applied before or after WOD enhanced functional capacity and decreased inflammatory, muscle damage, and oxidative stress biomarkers in CrossFit® athletes. Unlike the results associated with other exercise protocols, our results suggest that PBMT-sMF may be applied before or after WOD in CrossFit® athletes according to athletes' or therapists' preferences. Furthermore, the mixed outcomes observed when PBMT-sMF was applied before and after the WOD may be related to an overdose and not necessarily due to the intervention at these two moments.

One strength of this clinical trial was the blinding of the assessors, therapists, and participants. In addition, the trial was prospectively registered and followed without violation of the registered protocol. High methodological standards were strictly followed, including intention-to-treat analysis, allocation concealment, and true randomization. We also included a placebo group to address possible confounding factors. The sample size was based on an appropriate calculation to detect between-group differences in the primary outcome. The limitations of this study were the measurement of the effects of PBMT-sMF for up to 48 hours and the inclusion of only male volunteers.

Further studies with rigorous methodological quality and adequate sample size are needed to investigate the long-term effects of implementing PBMT-sMF in the daily routines of CrossFit® training and competition. Further studies investigating the effects of PBMT-sMF on different oxidative stress markers are needed to establish this therapy as an antioxidant agent and to elucidate the exact mechanisms of action of PBMT-sMF.

## 5. Conclusion

PBMT-sMF enhanced the performance of functional tests when applied before or after WOD, but not when applied before and after WOD. Furthermore, PBMT-sMF decreased the levels of biochemical markers of muscle damage, particularly when applied before and after WOD. In contrast, PBMT-sMF modulated the biochemical markers of inflammation, regardless of the time of application. PBMT-sMF decreased oxidative stress and increased antioxidant activity in CrossFit® athletes when applied before or after WOD. Therefore, in mixed activities, such as CrossFit®, it seems more effective to apply PBMT-sMF before or after exercise.

## Figures and Tables

**Table 1:** PBMT-sMF parameters.

	Knee extensors	Knee flexors	Plantar flexors
Number of lasers	4	4	4
Wavelength (nm)	905	905	905
Frequency (Hz)	250	250	250
Peak power (W)—each	50	50	50
Average mean optical output (mW)—each	1.25	1.25	1.25
Power density (mW/cm^2^)—each	3.91	3.91	3.91
Energy density (J/cm^2^)—each	0.50	0.44	0.44
Dose (J)—each	0.16	0.14	0.14
Spot size of laser (cm^2^)—each	0.32	0.32	0.32
Number of red LEDs	8	8	8
Wavelength (nm)	633	633	633
Frequency (Hz)	2	2	2
Average optical output (mW)—each	25	25	25
Power density (mW/cm^2^)—each	29.41	29.41	29.41
Energy density (J/cm^2^)—each	3.79	3.39	3.39
Dose (J)—each	3.22	2.88	2.88
Spot size of red LED (cm^2^)—each	0.85	0.85	0.85
Number of infrared LEDs	8	8	8
Wavelength (nm)	850	850	850
Frequency (Hz)	250	250	250
Average optical output (mW)—each	40	40	40
Power density (mW/cm^2^)—each	71.23	71.23	71.23
Energy density (J/cm^2^)—each	9.21	8.21	8.21
Dose (J)—each	5.16	4.60	4.60
Spot size of red LED (cm^2^)—each	0.56	0.56	0.56
Magnetic field (mT)	110	110	110
Irradiation time per site (sec)	129	115	115
Total dose per site (J)	67.68	60.76	60.76
Total dose applied per lower limb (J)	270.72	182.28	60.76
Aperture of device (cm^2^)	33	33	33
Application mode	Direct skin contact and slight pressure	Direct skin contact and slight pressure	Direct skin contact and slight pressure

**Table 2:** Absolute values about functional capacity, rating of perceived exertion (RPE), muscle damage, inflammatory activity, and oxidative stress (*n* = 12).

Variables	Intervention (*n* = 12)	Baseline (*n* = 12)	Immediately after the WOD (*n* = 12)	Immediately after the intervention (*n* = 12)	1 h (*n* = 12)	24 h (*n* = 12)	48 h (*n* = 12)
Performance on functional test (free squat reps)	Placebo	67.64 (±6.09)	—	62.09 (±8.15)	66.36 (±5.55)	66.45 (±6.98)	65.82 (±7.45)
	PBMT-sMF before	65.73(±7.32)	—	63.27 (±9.02)	66.18 (±5.31)	67.09 (±5.50)	67.73 (±6.15)
	PBMT-sMF after	63.09 (±8.04)	—	63.82 (±6.69)	65.27 (±6.53)	67.18 (±6.93)	67.45 (±6.64)
	PBMT-sMF before and after	65.91 (±6.82)	—	61.55 (±9.17)	65.64 (±6.31)	67.45 (±6.28)	67.45 (±5.20)
Exertion subjective perception (RPE cardiorespiratory)	Placebo	5.81 (±12.10)	80.00 (±27.84)	14.81 (±18.24)	8.54 (±13.99)	2.54 (±5.04)	1.92 (±4.35)
	PBMT-sMF before	1.31 (±1.11)	81.85 (±19.98)	13.31 (±13.46)	2.38 (±4.44)	3.00 (±6.36)	2.52 (±8.27)
	PBMT-sMF after	3.85 (±5.96)	84.23 (±19.88)	15.92 (±21.41)	4.77 (±88.89)	1.15 (±3.00)	1.00 (±2.83)
	PBMT-sMF before and after	5.00 (±7.70)	78.20 (±26.96)	16.80 (±21.18)	7.60 (±12.06)	2.00 (±6.32)	0.70 (±1.64)
Exertion subjective perception (RPE muscles)	Placebo	9.27 (±8.81)	81.92 (±29.08)	27.92 (±23.85)	10.58 (±11.35)	12.19 (±11.79)	11.38 (±15.98)
	PBMT-sMF before	5.27 (±8.37)	91.54 (±17.37)	24.77 (±20.00)	13.54 (±20.01)	12.77 (±11.59)	9.22 (±12.87)
	PBMT-sMF after	10.35 (±15.00)	90.38 (±15.34)	26.85 (±19.78)	16.25 (±15.89)	7.35 (±6.88)	6.85 (±14.18)
	PBMT-sMF before and after	9.20 (±15.33)	82.00 (±20.44)	33.30 (±26.22)	11.45 (±12.95)	8.50 (±11.65)	5.75 (±6.49)
Muscle damage (CK (l*μ*/l))	Placebo	145.29 (±115.80)	—	—	170.52 (±130.38)	207.93 (±167.92)	198.94 (±192.23)
	PBMT-sMF before	158.60 (±127.39)	—	—	168.70 (±123.27)	189.04 (±157.47)	127.65 (±104.57)
	PBMT-sMF after	153.10 (±120.34)	—	—	163.01 (±125.30)	170.74 (±115.94)	110.45 (±99.73)
	PBMT-sMF before and after	190.38 (±188.89)	—	—	199.21 (±209.30)	198.78 (±203.80)	145.16 (±149.84)
Inflammation (IL-6 (pg/ml))	Placebo	8.95 (±2.76)	—	—	13.43 (±2.88)	11.94 (±3.05)	12.78 (±3.93)
	PBMT-sMF before	8.16 (±1.51)	—	—	10.68 (±1.80)	7.79 (±1.44)^aa^	9.38 (±2.17)^a^
	PBMT-sMF after	8.76 (±2.50)	—	—	12.43 (±3.43)	8.08 (±2.72^)aa^	10.24 (±2.55)
	PBMT-sMF before and after	8.58 (±2.06)	—	—	12.77 (±3.24)	8.25 (±2.04)^aa^	9.75 (±2.59)^a^
Oxidative stress (TBARS) (nmol/ml)	Placebo	3.20 (±0.28)	—	—	4.38 (±0.37)	5.41 (±0.64)	6.66 (±0.17)
	PBMT-sMF before	3.36 (±0.33)	—	—	4.57 (±0.30)	5.44 (±0.26)	4.61 (±0.31)^aaaabbbb^
	PBMT-sMF after	3.32 (±0.18)	—	—	4.60 (±0.32)	5.53 (±0.33)	4.68 (±0.30)^aaaabbbb^
	PBMT-sMF before and after	3.46 (±0.38)	—	—	4.49 (±0.34)	5.52 (±0.29)	6.45 (±0.35)
Oxidative stress (carbonylated proteins) (nmol of DNPH/g/dl of protein)	Placebo	4.97 (±0.55)	—	—	5.98 (±0.32)	6.52 (±0.72)	6.73 (±0.64)
	PBMT-sMF before	5.22 (±0.21)	—	—	5.94 (±0.36)	5.66 (±0.50)^aabb^	5.76 (±0.54)^aaaabbbb^
	PBMT-sMF after	5.12 (±0.40)	—	—	5.91 (±0.36)	5.62 (±0.22)^aabb^	5.81 (±0.50)^aa bbbb^
	PBMT-sMF before and after	5.01 (±0.35)	—	—	6.14 (±0.53)	6.55 (±0.64)	6.93 (±0.66)
Antioxidant activity (CAT) (U CAT/g of protein)	Placebo	4.39 (±0.56)	—	—	4.60 (±0.40)	3.67 (±0.41)	3.27 (±0.21)
	PBMT-sMF before	4.41 (±0.58)	—	—	5.07 (±0.32)^a^	5.28 (±0.43)^aaaabbbb^	5.27 (±0.19)^aaaabbbb^
	PBMT-sMF after	4.37 (±0.40)	—	—	5.06 (±0.45)^a^	5.36 (±0.47)^aaaabbbb^	5.19 (±0.23)^aaaabbbb^
	PBMT-sMF before and after	4.18 (±0.52)	—	—	4.66 (±0.39)	3.54 (±0.42)	3.38 (±0.25)
Antioxidant activity (SOD) (U SOD/g of protein)	Placebo	3.36 (±0.34)	—	—	3.45 (±0.52)	3.04 (±0.46)	2.91 (±0.30)
	PBMT-sMF before	3.50 (±0.48)	—	—	3.61 (±0.63)	4.01 (±0.38)^aaaabbbb^	4.12 (±0.34^aaaabbbb^
	PBMT-sMF after	3.44 (±0.36)	—	—	3.55 (±0.41)	3.97 (±0.31)^aaaabbb^	4.32 (±0.54)^aaaabbbb^
	PBMT-sMF before and after	3.27 (±0.29)	—	—	3.32 (±0.55)	3.20 (±0.51)	2.89 (±0.24)

Values are expressed as the mean ± standard deviation (±SD). Compared to placebo: ^a^*p* < 0.05, ^aa^*p* < 0.01, and ^aaaa^*p* < 0.0001. Compared to PBMT-sMF before and after WOD: ^bb^*p* < 0.01, ^bbb^*p* = 0.001, and ^bbbb^*p* < 0.0001.

## Data Availability

The datasets generated and analyzed during the current study are available from the corresponding author on reasonable request.
